# Pointing Out Some Issues Regarding Reproduction Management in Murciano-Granadina Goats

**DOI:** 10.3390/ani11061781

**Published:** 2021-06-15

**Authors:** Nemesio Fernández, M. Carmen Beltrán, Gema Romero, M. Amparo Roca, Martín Rodríguez, Sebastián Balasch

**Affiliations:** 1 Instituto de Ciencia y Tecnología Animal, Universitat Politècnica de València, Camino de Vera, s/n, 46022 València, Spain; mbeltran@dca.upv.es (M.C.B.); mrodriguez@dca.upv.es (M.R.); 2 Centro de Investigación e Innovación Agroalimentaria y Agroambiental, Universidad Miguel Hernández, Orihuela, Carretera de Beniel, Km. 3,2, 03312 Orihuela, Spain; gemaromero@umh.es; 3 Escuela Politécnica Superior de Orihuela, Universidad Miguel Hernández, Orihuela, Carretera de Beniel, Km. 3,2, 03312 Orihuela, Spain; aroca@umh.es; 4 Departamento de Estadística e Investigación Operativa Aplicadas y Calidad, Universitat Politècnica de València, Camino de Vera, s/n, 46022 València, Spain; sbalasch@eio.upv.es

**Keywords:** progesterone, milk, reproduction, goat, Murciano-Granadina breed

## Abstract

**Simple Summary:**

The hypothesis of this experiment proposes that it could be possible to identify pregnant goats through maximum progesterone milk levels at any time in the pregnancy, and that there is an optimal moment to apply a lactation inhibitor to dry off lactating goats. The maximum progesterone concentration in milk varied depending on the season of the year, and those concentrations were similar for pregnant and non-pregnant goats, but significantly higher in the case of gestating goats with four foetuses, for which it would be possible to distinguish the pregnancy. The milk yield of goats at mating does not affect fertility until a value of at least 3250 mL/day. If using lactation inhibitors, their application up to the 10th week post-mating would be optimal for drying off lactating goats.

**Abstract:**

Two of the most important problems in high-yielding dairy goat farms are early and accurate pregnancy diagnosis and the appropriate dry off of lactating does before the next kidding. The hypothesis posits that it could be possible to identify pregnant does through maximum progesterone milk levels at any time during the pregnancy, and that there is an optimal time to apply a lactation inhibitor to help dry off lactating does. Therefore, 114 Murciano-Granadina breed goats were used, from which 74 goats were inseminated at week 20 of lactation and samples of milk from pregnant and non-pregnant goats were taken at two-week intervals. The average maximum progesterone milk levels were higher outside the natural breeding season (40° latitude) than in the breeding season (11.6 ± 1.13 vs. 8.6 ± 1.02 ng/mL), although the levels from pregnant and non-pregnant goats were similar (10.85 ± 1.3 vs. 9.74 ± 1.6 ng/mL), except in the case of pregnancy with four foetuses (12.5 ± 1.3 ng/mL). Milk yield at mating does not affect fertility until a value of at least 3250 mL/day. Pregnancy started to affect milk yield up to the +7th week and was 59.9% lower in the +10th week after mating, so the use of lactation inhibitors could be more effective from this latter week. In conclusion, the results show that it is not possible to detect gestation in goats reliably through the maximum concentration of progesterone in milk at any time during lactation, except in the case of goats gestating four foetuses, that the milk yield of goats at mating does not affect fertility until a value of at least 3250 mL/day, and that from the 10th week post-mating, the application of lactation inhibitors would be optimal.

## 1. Introduction

Two of the most important problems in high yielding dairy goat farms are early and accurate pregnancy diagnosis [1] and the appropriate dry off of lactating does before the next kidding [2]. On one hand, some authors [3,4] have discussed and classified the pregnancy diagnosis methods, whose choice depends on the delay in post-breeding before the animals are examined, equipment availability and the desired accuracy, with ultrasonography as one of the most reliable methods [4], although measuring the progesterone concentration in blood and milk is becoming increasingly common [1]. The progesterone concentrations in blood and milk follow the same pattern; the concentrations of the cycle are very low from day 1 to day 3, increasing from day 4 to day 12 (the size of the corpus luteum increases considerably), and staying similar until days 16–18, then falling rapidly 2–4 days before oestrus [5]. By measuring the progesterone concentration in a blood or milk sample, drawn between 19 and 23 days after breeding, non-pregnant does may be reliably identified by their low progesterone values, whereas when concentrations are high, the probability of pregnancy is around 90% [6]. Regarding the absolute values, concentrations of progesterone in milk are much higher than in plasma [7,8], due to the passage of the hormone through the blood circulation in the mammary gland [9], to its affinity for fatty esters and its ability to bind to carrier proteins [10,11]. Thus, progesterone concentrations in milk above 10 ng/mL from 22 and 26 days after breeding correspond to goats classified as positive for pregnancy, although these concentrations may vary depending on the time of year [12]. Individual samples from pregnant and non-pregnant females at different times of the oestrous cycle arrive at the laboratories that analyse milk from dairy records from herds under genetic selection programmes, so the progesterone concentrations may be higher for pregnant goats than during the luteal phase of non-fertile oestrous cycles, as occurs in cows [13]. It is hypothesised that the maximum levels of progesterone in milk could be different between pregnant and non-pregnant goats during the luteal phase, which allows them to be distinguished at any point of the lactation when routinely analysing samples from herds under genetic selection programmes.

On the other hand, from Zobel et al. [2] it can be deduced that another of the greatest problems for dairy goat farms is the difficulty in drying off lactating goats before the next kidding. As the Murciano-Granadina (MG) goat breed are frequently used to one milking per day [14], for instance, the technique of reducing the number of milkings does not provide the desired effects. Thus, to achieve the drying off of the animals, in addition to the classic management techniques, certain lactation inhibitors have been used, such as bromocriptine and cabergoline [15,16]. However, the effect of each application of cabergoline only lasts 5–10 day in blood [17], so it is necessary to perform repeated applications [17] which, added to the possible effects of pregnancy, help to achieve a good dry off. According to Knight and Wilde [18], pregnancy begins to have an effect on the lactation curve of goats from the 8th week after mating, while Salama et al. [19] indicate that this date is delayed to the 10th week. In order to achieve a real symbiotic effect of the application of lactation inhibitors with the depressor effects of gestation on the drying off of goats, this study attempts to check the moment at which pregnancy has an important impact on the lactation curve of MG goats.

Therefore, this study aims to verify the possibility of identifying pregnant goats through maximum progesterone milk levels at any time in the pregnancy and propose an optimal time to apply a lactation inhibitor to dry off lactating goats.

## 2. Materials and Methods

### 2.1. Ethics

The housing and handling of the experimental animals followed the mandatory principles for the care and use of experimental animals in Spain (Real Decreto 53/2013, Boletín Oficial del Estado 34, 11370–11421). In this experiment, no manipulations of the animals typified as procedures were carried out.

### 2.2. Goats and General Proczedures

One hundred and fourteen multiparous (2.6 ± 0.3 years) healthy MG breed goats, weighing on average 46 ± 2 kg, were used at the experimental farms of the Spanish Universitat Politècnica de València (UPV; 39.5° north latitude; 52 goats) and Universidad Miguel Hernández (UMH; 38.1° north latitude; 62 goats). Thirty-seven goats from each university were inseminated with sperm from MG males and the others (15 goats from UPV and 25 goats from UMH) remained without treatment, as the control. Mating was synchronised using intravaginal sponges (30 mg fluorogestone acetate, Chrono-gest^®^, CEVA Salud Animal, Barcelona, Spain) in February (UPV) and May (UMH), injected with 450 International Units (IU; PMSG, CEVA Salud Animal, Barcelona, Spain) and inseminated at 20 weeks of lactation. For goats bred near to 40° north latitude, the breeding season starts in June and ends in March. Goats were milked once a day in a medium line milking parlour (UPV and UMH). All goats included in this work were diagnosed as free from mastitis throughout the experiment.

### 2.3. Experimental Data and Sample Collection

Actual milk (milked milk) was recorded from the 14th to 32nd week of lactation, every other week, at 0800 h on Tuesday, from December to May (UPV) and from March to August (UMH), and samples (50 mL) of total actual milk were collected and immediately analysed for milk composition and Somatic Cell Count (SCC). Milk composition (fat, protein, lactose and non-fat solids (NFS)) was analysed with a medium infrared analyser (Milkoscan^®^ FT6000; Foss Iberia, Barcelona, Spain) and SCC was determined by the fluoro-opto-electronic method (ISO, 2008; Fossomatic^®^ 5000, Foss Iberia, Barcelona, Spain). For the progesterone analysis, the milk samples were preserved with sodium azide tablets (Merck KGaA, Darmstadt, Germany), frozen at −40 °C, and sent, immediately after freezing, to the laboratories of the Centro de Investigación y Tecnología Agroalimentaria (CITA) of Aragón. Seventy-six of these samples were in duplicate. Progesterone concentration in goat’s whole milk was determined using a direct competitive-binding ELISA commercial kit suitable for the bovine, ovine and caprine species (Milk ELISA 1.0 Progesterone; Ridgeway Research Ltd., St Briavels, Gloucestershire, UK) following the manufacturer instructions.

### 2.4. Statistical Analysis of Results

A principal components analysis (PCA) was used to identify the main components of the global variability corresponding to the set of seven variables studied (actual milk, concentration of progesterone in milk, fat content, protein content, lactose content, NFS and SCC). A variance components analysis was used to decompose the variability of progesterone values with a nested model of two factors: animal and record (animal), on a total of 152 samples analysed in duplicate. A couple of *T*-test analyses were used to compare two means between independent observations of actual milk at the moment of mating, on one hand, and of the maximum level of progesterone in milk after mating, on the other hand, for the groups of pregnant and non-pregnant goats. To see the effects of the season of the year on the mean minimum and maximum progesterone values in milk, and the number of kids born on the mean progesterone levels in milk and on actual milk after mating, One-Way ANOVA analyses were carried out, with the corresponding comparisons of means performed through the Tukey test. To study the bi-weekly evolution of actual milk, level of progesterone in milk, fat, protein and lactose milk contents, and SCC, a General Linear Model was used to analyse a repeated measures design that included the fixed effects of reproductive status (pregnant or non-pregnant), week of lactation and their interaction, the random effect of the animal (nested in reproductive status) and residual error. The SCC logarithm (logSCC) was used to normalise SCC distribution [20]. The results were analysed using Statgraphics Centurion software (version XVI.II). Tukey’s confidence intervals directly show the existence of significant differences when there is no overlap between those intervals and vice versa.

## 3. Results

### 3.1. Principal Components

In the PCA, component 1 explained 46.4% of the variance, while components 2 and 3 explained 15.5% and 12.1%, respectively, which means that between the three together explained 74.0% of the global variability corresponding to the set of seven variables studied (actual milk, progesterone content in milk, fat content, protein content, lactose content, NFS and logSCC).

**Figure 1:**
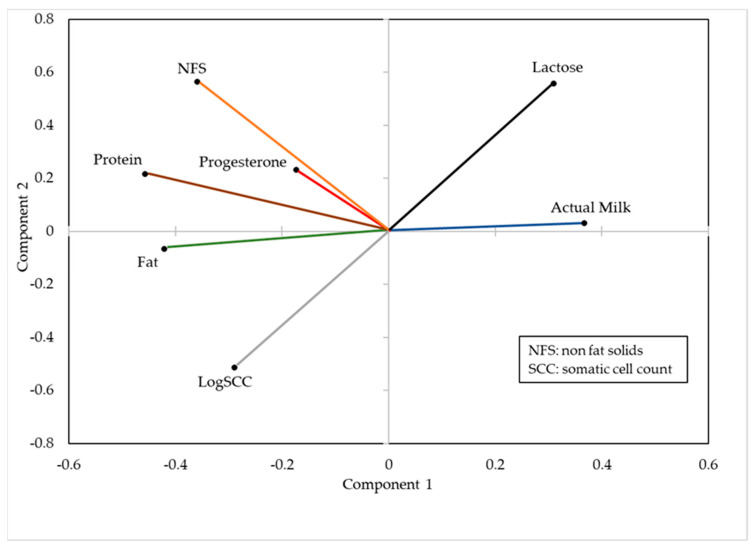
Variable coefficients that make up the principal components 1 and 2 (*n* = 786).

The coefficients of principal components 1 and 2 are presented in Figure 1. The positive relationship between the coefficients of actual milk and lactose content, and the strong antagonism between the coefficients of lactose levels in milk and the logSCC, can be verified. On the other hand, actual milk was opposite to the concentrations of fat and protein, while the levels of NFS and the concentration of progesterone were in a second opposition level.

**Figure 2:**
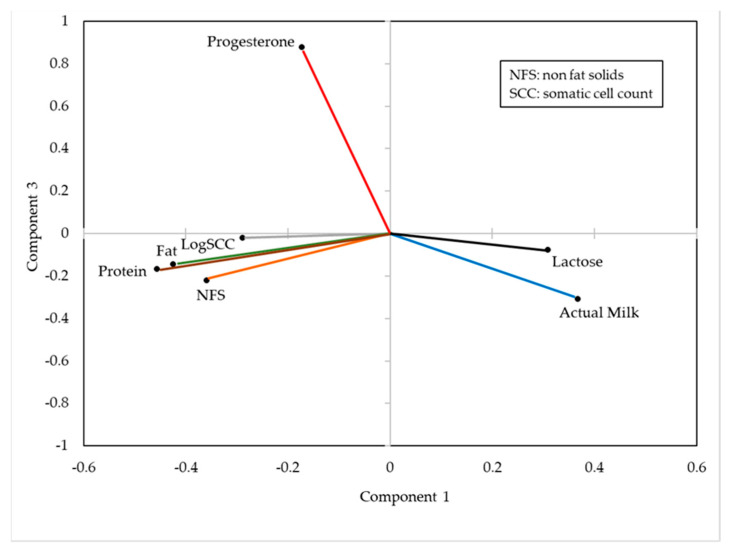
Variable coefficients that make up the principal components 1 and 3 (*n* = 786).

The coefficients of the principal components 1 and 3 are presented in Figure 2, which allows us to observe that this latter component clearly basically refers to the concentration of progesterone in milk.

### 3.2. Components of Variance for Progesterone Values in Milk

Table 1 shows the weight of the different variation factors on the concentration of progesterone in milk samples analysed in duplicate, highlighting the individual effect (goat) and the lactation status for their relevance. A very small variance component corresponded to the “others” section, in which a series of uncontrolled factors were grouped, among them the variability associated with the analytical procedure.

### 3.3. Progesterone Concentrations in Milk

The mean minimum and maximum progesterone concentrations in milk at different seasons of the year are presented in Table 2. The minimum mean values were below 1.5 ng/mL and similar at any time of the year. However, the maximum mean values were similar in winter and spring, and higher than those obtained in the most natural breeding seasons in this geographical latitude (around 40° north), such as summer and autumn, and at the same time, they were also similar in their maximum progesterone concentrations.

**Figure 3:**
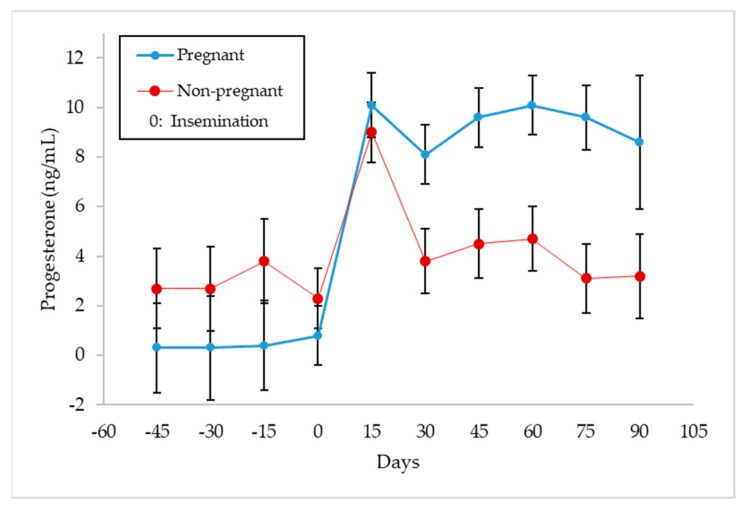
Evolution of milk progesterone concentration (ng/mL ± Tukey intervals) for pregnant and non-pregnant goats (*n* = 793).

Figure 3 shows the evolution of the mean concentration of progesterone in milk for pregnant and non-pregnant goats throughout the lactation period studied (*p* < 0.0001). It can be seen that prior to artificial insemination (−45 to 0 days), the mean progesterone concentration in the milk of goats that were going to become pregnant was 0.30 ± 2.1 ng/mL, whereas it was 2.88 ± 1.5 ng/mL for goats that would remain non-pregnant. After artificial insemination (0 to +90 days), the mean values of progesterone were higher (9.34 ± 1.6 vs. 5.47 ± 1.4 ng/mL; *p* < 0.0001) for pregnant animals. The progesterone concentration obtained at +15 days post-insemination went up, which is similar to the mean value of the following records for pregnant goats (10.1 ± 1.2 vs. 9.2 ± 1.6 ng/mL), while it is much higher in the case of non-pregnant goats (9.0 ± 1.1 vs. 3.86 ± 1.4 ng/mL).

**Figure 4:**
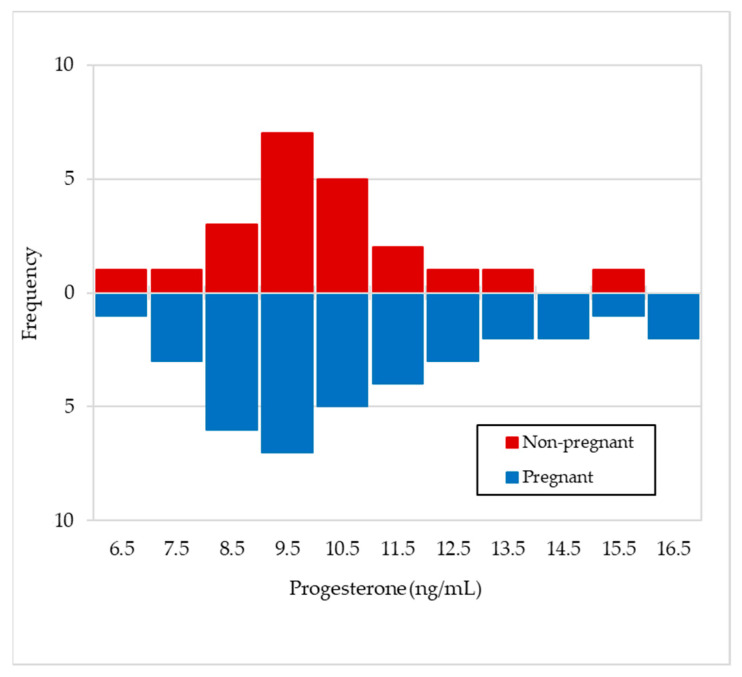
Frequency of maximum progesterone mean values in milk after mating for pregnant (*n* = 36) and non-pregnant goats (*n* = 22).

The number of goats that showed different mean maximum progesterone values in milk after the day of mating (day 0), depending on whether they were pregnant or not, are presented in Figure 4. A similar distribution can be seen in both cases, and mean values of 10.85 ± 1.3 and 9.74 ± 1.6 ng/mL, respectively, with statistically non-significant differences (*p* = 0.336), were obtained.

### 3.4. Relationship between Milk Production and Pregnancy

**Figure 5:**
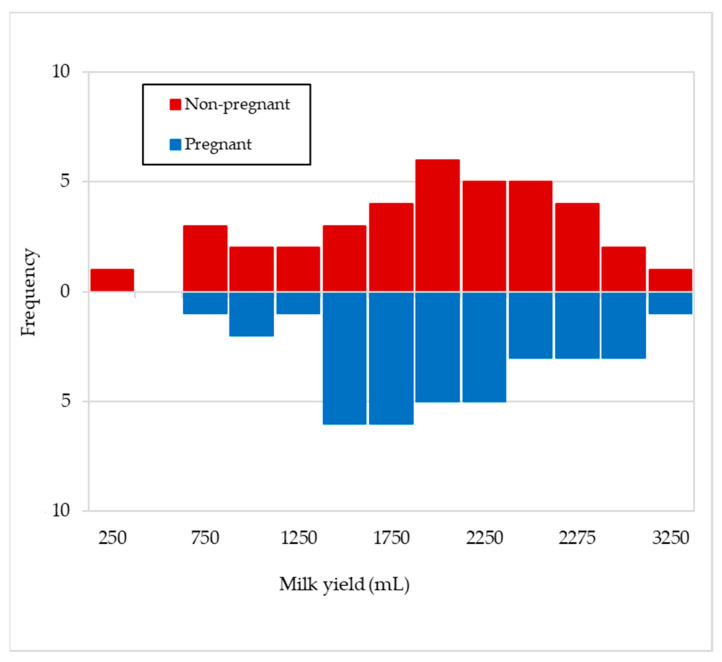
Frequency of pregnant (*n* = 36) and non-pregnant (*n* = 38) goats depending on actual milk at mating.

The frequencies of pregnant or non-pregnant goats depending on the average level of actual milk on the day of mating (day 0) are presented in Figure 5. It can be seen that the distribution of pregnant and non-pregnant is similar for the entire actual milk range studied (250–3250 mL/day). In fact, the distribution of the 74 goats included in this case was 36 pregnant and 38 non-pregnant.

**Figure 6:**
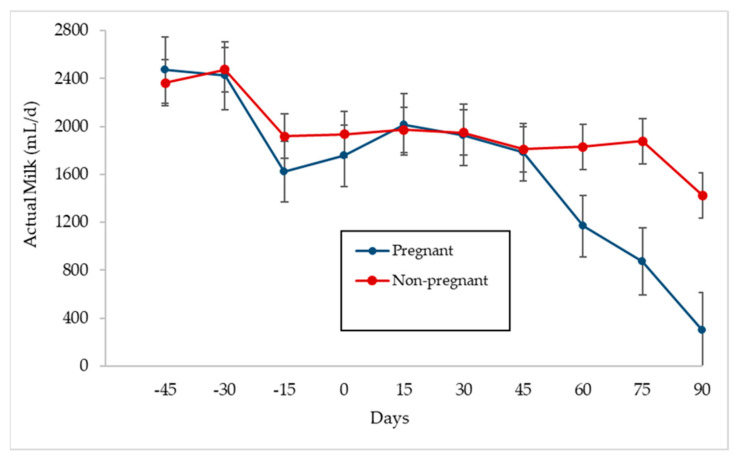
Actual milk evolution (mL/day ± Tukey intervals) for pregnant (*n* = 36) and non-pregnant goats (*n* = 78) during the studied lactation period.

The evolution of the mean actual milk depending on whether the goats were pregnant or non-pregnant is presented in Figure 6. This figure shows that from day +45 of pregnancy, there was a significant (*p* < 0.0001) decrease in milk production, so that production drops were 34%, 25.5% and 66% on days +60, +75 and +90 post-insemination, respectively, compared to the previous records, which implies a drop in milk production until the moment of drying off (between +45 and +90 days after insemination) of 1489 mL/day (83%), whereas it was 385 mL/day (21%) for the group of non-pregnant goats.

### 3.5. Effect of the Number of Kids Born

There were no significant differences of pregnancy duration depending on the number of kids born (Table 3).

**Figure 7:**
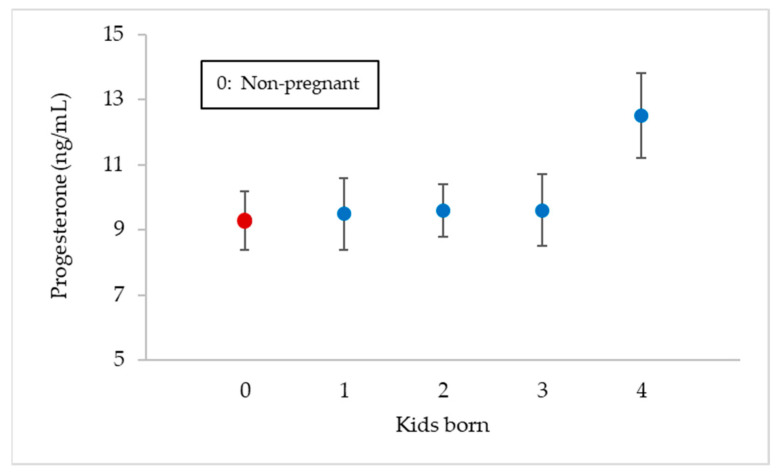
Milk progesterone maximum mean values (ng/mL ± Tukey intervals) between days +15 and +90 post-mating for goats non-pregnant (*n* = 78) and for those that gave birth to one, two, three or four kids (*n* = 36).

Figure 7 represents the mean progesterone values in milk from day +15 post-insemination to the end of lactation (+90 day) depending on the number of kids gestated. It can be seen that there are significant differences (*p* < 0.033) between the progesterone concentration of goats that gave birth to four kids (12.5 ± 1.3 ng/mL) and those of non-pregnant goats (9.3 ± 0.9 ng/mL), as well as the ones that gave birth to one (9.5 ± 1.1 ng/mL), two (9.6 ± 0.8 ng/mL) or three kids (9.6 ± 1.1 ng/mL).

**Figure 8:**
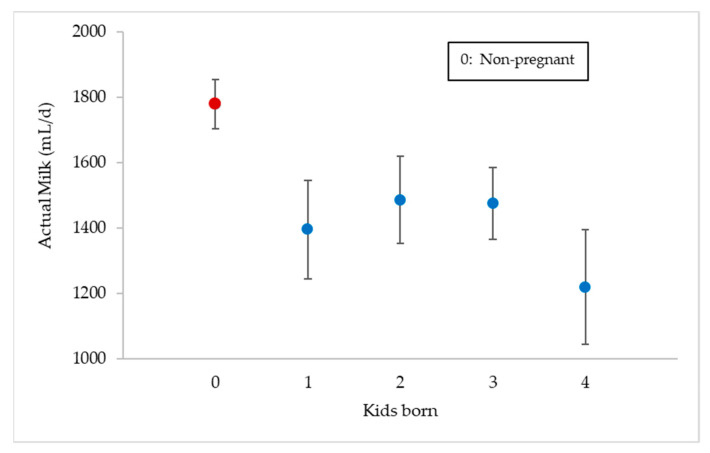
Actual milk mean values (mL/day ± Tukey intervals) for non-pregnant goats (*n* = 78) and for those that gave birth to one, two, three or four kids (*n* = 36) between days +15 and +90 post-insemination.

On the other hand, Figure 8 represents the mean actual milk values from day +15 post-insemination to the end of lactation (+90 day) depending on the number of kids born. It can be seen that there were significant differences (*p* < 0.0001) between non-pregnant goats (1779 ± 75 mL/day) and those that gave birth to one (1395 ± 150 mL/day), two (1485 ± 133 mL/day), three (1475 ± 110 mL/day) or four kids (1219 ± 175 mL/day). 

### 3.6. Effect of Pregnancy on Milk Components

From +45 day of pregnancy, there was a significant (*p* < 0.0001) increase in milk fat (37%), milk protein (49%) and milk logSCC (17%) to +90 day after insemination, and a significant (*p* < 0.0001) decrease in lactose (10%), whereas for non-pregnant goats, the evolutions were: −1.7% for fat and −1.0% for protein, lactose and logSCC. 

## 4. Discussion

An analysis of the relationships among the different variable coefficients studied in this experiment through a PCA, as well as the verification of the reliability of the methodology used for the analysis of progesterone, indicates, in a general way, that the results obtained are in agreement with those reported by other authors. Thus, the fact that there is a positive relationship between milk yield and the lactose concentration in milk or that a higher quantity of milk produced leads to a reduction in concentrations of fat and protein are in agreement, among others, with [21,22,23]. Moreover, the contrast of concentrations between lactose and SCC in milk agrees with the observations made by Leitner et al. [24] and Stocco et al. [25]. The results also show that, in general, in the analysis of the data of this paper, it should be noted that when the concentration of progesterone in milk increases, the rest of the variables analysed tend to decrease. The fact that the “other” factor of the variance components of the progesterone values represents only 4.35% indicates a high reliability of the analytical approach used.

Fertility by artificial insemination was 49% (36 goats pregnant from 74 inseminated), similar to the 47.9% obtained by Alabart et al. [26] in ewes and outside the natural breeding season, and prolificacy was 2.14, similar to the 2.1 obtained by Pérez-Baena et al. [27]. Regarding the effect of the level of actual milk and the possibility of the goat becoming pregnant, Figure 5 shows that the frequency distribution of pregnant and non-pregnant goats was similar, at least until 3250 mL/day. Nebel and McGuilliard [28] state that the demands of high milk yield can compromise reproductive performance due to delayed ovarian activity and reduced conception rates, something that did not occur in this experiment up to the stated production level. 

In this work, it is verified (Table 2) that the minimum values of progesterone corresponding to cyclic animals are similar at any time of the year, while the maximum values are higher outside the natural breeding season (winter and spring) in the latitude at which these experiments were carried out (around 40° north). These results do not agree with those obtained by van Binsbergen et al. [29] in cows, who reported maximum values of 15 ng/mL of progesterone in milk in the month of May, while those values rose between 20–25 ng/mL for the months of June and July. Nor do they agree with those of Blaszczyk et al. [30] in goats, who obtained results significantly higher (*p* < 0.01) for autumn (breeding season) than in spring (outside the breeding season) at both basal (0.50 ± 0.15 vs. 0.25 ± 0.04 ng/mL) and mean concentrations from the whole observation period (0.42 ± 0.11 vs. 0.27 ± 0.08 ng/mL) of progesterone in plasma. Perhaps the lower relationship between the acceptance of mounting and the time of year in the case of cows [30], compared to what occurs in goats [27], could help explain the divergence of the results between species. In addition, an interference between lactation and reproductive periods could take place. Therefore, in autumn, the goats of the UPV were near the peak of lactation and, therefore, in a state of low body condition score that could lead to a lower number of goats coming into heat, and thus accounts for a lower concentration of progesterone in milk than expected. On the contrary, the same goats were in mid or late lactation in winter and autumn, with milk production figures lower than those observed in autumn and, therefore, with a better body condition score, which favours coming into heat and higher concentrations of progesterone in milk than expected. 

In Figure 3, it can be observed that the mean progesterone values during the days prior to insemination (0 to −45 days) varied between 0.1–0.8 ng/mL for the goats that were going to become pregnant, which seems to indicate that there are very few cyclic animals. However, those goats that were not going to become pregnant did so between 2.3–3.8 ng/mL, which could indicate the existence of some cyclical animals and/or with some follicular disorder [31,32]. The mean progesterone values rose on day +15, indicating the presence of a functional corpus luteum (CL), and these values continued at the same level until the end of lactation in the case of pregnant goats, which makes sense, as the CL is the only source of progesterone for the maintenance of pregnancy in goats [33]. One of the aims of knowing the progesterone values in milk after mating focuses on the early detection of the physiological state of the animals (pregnant or non-pregnant); therefore, in goats, samples should be collected on designated days (+17–20 days post-mating) [26] in which low values would be indicative of a cyclical animal, whereas high values would indicate a gestation. Thus, Jain et al. [34], Engeland et al. [35], González-Stagnaro et al. [36], de Castro et al. [37] and Górecky et al. [38], in cyclic goats, found baseline progesterone values between 0–0.629 ng/mL and maximum values between 6–12.7 ng/mL, while Dawson [39] obtained a threshold of 0.94 ng/mL of progesterone in plasma on day +20 after mating, to distinguish pregnant from non-pregnant goats. In relation to this, Stewart and Shipley [40] indicate that progesterone concentrations can be measured in milk or serum and collected precisely one cycle after the goat was bred, but whereas low progesterone levels can confirm a non-pregnant status, high progesterone is not a positive pregnancy test, as it cannot differentiate between mid-cycle, true pregnancy or false pregnancy. On the same line, Bretzlaff et al. [12] and Fleming et al. [41] indicate that milk progesterone concentration varies from day to day and also with the type of milk sample obtained (foremilk, mid-milk and strippings) and with milk yield levels in goat, whereas plasma progesterone concentrations tend to be more accurate than milk, so that progesterone testing in goats is a good test for non-pregnancy, but only a fair test for pregnancy. Thus, Dawson [39] indicates that milk progesterone levels in goats ≥10 ng/mL on days 22–26 after mating indicate pregnancy with accuracy >86%, whereas concentrations ≤10 ng/mL denote a lack of pregnancy with up to 100% accuracy. Regarding the observations of progesterone in blood or milk, Pennington et al. [42] state that it has been shown to vary in a similar pattern throughout the oestrous cycle in goat, while Mann et al. [13] always obtained higher values in milk than in plasma. However, it is interesting to enhance some type of discernment of the reproductive status of animals through individual milk samples that reach the laboratories involved in genetic improvement programmes, as they would be an important complement to the usual information on concentrations of milk fat, protein, lactose, SCC or bacteriology in milk. For this reason, it would be very useful to know whether individual milk samples obtained at any time post-mating could contain maximum progesterone values that were markedly different between pregnant animals and those in the luteal phase of a cyclic animal. For example, in the case of cattle, Mann et al. [13] obtained maximum progesterone concentration significantly (*p* < 0.05) lower in non-pregnant cows than in pregnant ones from day 12 post-insemination (20 vs. 14 ng/mL in milk and 9 vs. 6 ng/mL in plasma). However, the fact that, in this work (Figure 3), non-pregnant goats presented that a mean concentration of progesterone in milk significantly lower than that of pregnant goats is not sufficiently valid to discern their reproductive status, as it simply indicates mean values of cyclic goats are not synchronised (not pregnant) compared to those of pregnant females. In fact, the similar distribution of maximum progesterone concentration values in milk between pregnant and non-pregnant animals (Figure 4) seems to indicate that this possibility is not feasible (10.85 ± 1.3 and 9.74 ± 1.6 ng/mL, respectively). This discernment would only be possible in the case of comparing the concentrations of non-pregnant goats vs. pregnant goats with four foetuses (9.74 ± 1.6 and 12.50 ± 1.3 ng/mL, respectively; Figure 7). Both in this experiment and in that of Pérez-Baena et al. [27], a mean prolificacy of 2.1 was observed in MG goats. Therefore, an average ovulation of two to three oocytes for non-pregnant goats in this experiment is expected and, therefore, two or three active corpora lutea, which could explain the results obtained in our case. Other authors [43,44,45] found significant differences (*p* < 0.01) in the concentration of progesterone in milk from goats carrying one foetus vs. those carrying two or three foetuses (19.2–29.9 vs. 9.2 ng/mL, respectively), after the second half of pregnancy. The explanation for all of this would be because, as already mentioned, the CL is the only source of progesterone for the maintenance of pregnancy in goats, so an increase in CL number may contribute more progesterone secretion [46]. 

On the other hand, one of the problems of dairy goat farms is the difficulty in drying off the goats prior to the next parturition and lactation [47]. Vilar and Rahala-Schultz [47] indicate several drying practices that usually include various milk cessation methods: restricted feeding, reducing the milking frequency, the application of internal teat sealants and the administration of antibiotic dry therapy, some of which, according to Caldeira et al. [48], could increase the risk of ketone bodies toxaemia. In this work, it has been verified that from day +45 post-mating, there was a significant drop in milk production and that it tended to be higher in the case of gestations with four kids. In goats, Knight and Wilde [18] point out that the influence of pregnancy started to become significant after week 8 post-mating, while Salama et al. [19] indicate that this moment happened at week 10 of pregnancy. The drop in milk production begins from week 6 post-mating and a significant reduction in milk occurs at week 10, as in the case of Salama et al. [19] in the same breed, so to optimise the application of a possible lactation inhibitor, it should be applied from the latter. Roche [49], in cows, observed a significant decline in milk yield from day 147 of gestation (at 33 weeks of lactation) in twins, after which pregnant cows produced 0.8 kg/cow per day less milk than non-pregnant twins. Oltenacu et al. [50] and NRC [51] say that the milk production cost of pregnancy in cows occurs through a combination of increased foetal demands for energy from 190 days of gestation onwards and a hormonally mediated partitioning of nutrients away from milk production. 

The results for milk components in this experiment were similar to those reported by Salama et al. [19], who observed, between week 2 and 12 of pregnancy, increases (*p* < 0.001) in milk fat, milk protein and logSCC, whereas milk lactose decreased (*p* < 0.001). On the other hand, Roche [49] observed that the yield of milk fat and protein was not affected by pregnancy until day 168 of gestation, after which pregnant cows produced 0.06 kg/cow per day less milk fat and 0.04 kg/cow per day milk protein compared with their non-pregnant twins. 

## 5. Conclusions

This work has studied several traits about the reproduction of Murciano-Granadina breed goats. It is verified that it is not possible to confirm the pregnancy of goats by the maximum value of progesterone in milk at any time during lactation, except in the case of goats gestating four foetuses, that actual milk of goats at mating does not affect fertility until a value of at least 3250 mL/day, and that in pregnant goats that are to be subjected to a drying off process with lactation inhibitors, it seems that the optimal time would be up to the 10th week of pregnancy.

## Figures and Tables

**Table 1:** Relevance (%) of variance components on progesterone concentration in milk (*n* = 152).

**Progesterone**	**Variance Component**		
	**Goat**	**Lactation Period**	**Others**
	65.08	30.56	4.35

**Table 2:** Mean minimum and maximum progesterone concentrations in milk (ng/mL ± Tukey intervals) at different seasons of the year.

Progesterone	Season of the Year				*p*-Value
	Spring	Summer	Autumn	Winter	
Minimum (*n* = 199)	1.18 ± 0.09	1.15 ± 0.15	1.27 ± 0.17	1.40 ± 0.18	*p* = 0.128
Maximum (*n* = 115)	11.6 ^a^ ± 1.10	8.85 ^b^ ± 0.91	8.28 ^b^ ± 1.12	11.56 ^a^ ± 1.15	*p* = 0.0006

^a,b^ Different superscripts in the same row indicate significant differences at *p* < 0.05.

**Table 3:** Duration of pregnancy (days ± Tukey intervals) depending on the number of kids born (*n* = 36).

**Pregnancy Duration**	**Number of Kids Born**				***p*-Value**
	**1**	**2**	**3**	**4**	
	147.2 ± 3.2	144.0 ± 2.4	146.3 ± 3.5	144.3 ± 3.4	*p* = 0.421

## Data Availability

The data is not available to the general public at this time.
